# Neuromuscular complications following targeted therapy in cancer patients: beyond the immune checkpoint inhibitors. Case reports and review of the literature

**DOI:** 10.1007/s10072-020-04604-1

**Published:** 2020-08-11

**Authors:** Chiara Demichelis, Andrea Balestra, Caterina Lapucci, Angela Zuppa, Stefano G Grisanti, Valeria Prada, Giampaola Pesce, Ilaria Grasso, Paola Queirolo, Angelo Schenone, Luana Benedetti, Marina Grandis

**Affiliations:** 1 Department of Neuroscience, Rehabilitation, Ophthalmology, Genetics, Maternal and Child Health (DINOGMI), University of Genoa, Genoa, Italy; 2 IRCCS Ospedale Policlinico San Martino, Genoa, Italy; 3 Department of Internal Medicine, University of Genoa, Genoa, Italy; 4 Autoimmunity Laboratory DiMI, University of Genoa, Genoa, Italy

**Keywords:** Targeted therapies, Immune-related adverse events, BRAF and MEK inhibitors, Imatinib, Neuromuscular complications, Myasthenia gravis

## Abstract

**Introduction:**

In the last years, many new drugs have been developed targeting different oncology pathways, overall improving both quality of life and survival in several malignancies. However, the increase of those therapies is associated with novel toxicities, mainly immune-related adverse events (irAEs), never observed before. Different irAEs are now well characterized, and, among them, neuromuscular complications, following immune checkpoint inhibitor (ICPi) therapy, are increasingly studied and described. However, there are also neurological complications related to the use of other targeted therapies, less known and probably underestimated. Herein we describe two oncological patients who developed neuromuscular diseases after administration of targeted therapies, different from ICPi.

**Case reports:**

The first patient was treated with the combination of Vemurafenib and Cobimetinib, BRAF and MEK inhibitors, respectively, for a cutaneous melanoma. One year after the beginning of the combined treatment, she developed a sub-acute motor neuropathy with predominant cranial nerve involvement. She was successfully treated with methylprednisolone. The second patient received therapy with Imatinib, tyrosine kinase inhibitor and precursor of the targeted therapy, for a gastrointestinal stromal tumour. Few days after the first administration, he developed generalized myasthenia gravis with respiratory failure. Clinical remission was obtained with plasma-exchange, intravenous immunoglobulins and steroids.

**Discussion and Conclusion:**

We strengthen the relevance of neuromuscular complications which may occur long after treatment start or in patients receiving not only the latest ICPi but also “older” and apparently better-known targeted therapies. Also in the latter cases, an immune-mediated “off-target” pathogenic mechanism can be hypothesized, and consequences can be life threatening, if not promptly diagnosed and appropriately managed.

## Introduction

In the last few years, many new drugs have been developed targeting always more selected and specific oncology pathways, overall improving both quality of life and survival in several malignancies. These drugs are associated to significant lower number of classical chemotherapeutic drug-related adverse events (AE). However, the increasingly widespread use of these therapies has led to the appearance of novel toxicities, mainly immune-related adverse events (irAEs), never observed before. Different irAEs are now well characterized, and, among them, neurological complications following immune checkpoint inhibitor (ICPi) therapy are always being increasingly studied and described [1–4].

However, there are also neurological complications related to the use of other targeted therapies, which are probably underestimated and with a less manifest immunological mechanism.

The scope of our study is to describe two cases of oncological patients, who developed different neuromuscular diseases after the administration of targeted therapy, different from ICPi. The importance of the first case is the time interval between starting therapy and the onset of the irAE. In the second case, what is striking is that myasthenia gravis developed after Imatinib administration, which was never described so far. Therefore, those cases expand the spectrum of neuromuscular irAE.

## Clinical report

### Patient 1

This 51-year-old woman was diagnosed with a left leg melanoma and inguinal lymph nodes micro-metastases. She underwent surgical excision of the cutaneous lesion associated with inguinal lymphadenectomy and immediately afterwards began oral adjuvant therapy with Vemurafenib, a BRAF kinase inhibitor. Two years later, because of the finding of abnormal lateral cervical and axillary lymph nodes uptake on total body positron emission tomography (TB-PET), the patient began a combination treatment; indeed, Cobimetinib, a MEK (mitogen-activated protein kinase kinase) inhibitor, was associated to Vemurafenib.

However, 9 months after the beginning of the combined treatment, she developed moderate adverse reactions: hives on the face and chest and headache, which resolved with temporary suspension of therapy for 2 weeks and steroid and antihistamine medications.

Nevertheless, 2 months later, she developed bilateral facial weakness, which progressed over the next 6 weeks. These symptoms improved when the patient was treated with betamethasone for a concomitant sciatic pain, but when she stopped steroid medication, they worsened again and she was admitted to the hospital. The neurological examination showed diplopia in all directions of gaze and right eye ptosis with slight fatigability, but not responsive to ice pack test, eyelid myokymia, bilateral peripheral facial nerve palsy, weakness of toes extension, numbness in fingers and feet and hyporeflexia in all four limbs.

The differential diagnosis included a direct infiltration of cranial nerves by the tumour, neuro-immunological causes related to the targeted therapy or to the tumour itself. Myasthenia gravis, neuropathies and myositis are described as the most frequent neuromuscular AE related to targeted therapy, while Lambert-Eaton syndrome and paraneoplastic polyneuropathies can be an immune-mediated complication of a tumour.

Myositis was excluded because of clinical characteristics and normal creatine phosphokinase (CPK). Contrast brain and spine MRI did not show any abnormality. The patient underwent electrodiagnostic studies: repetitive nerve stimulation test was normal, while the nerve conduction study revealed a motor neuropathy with low cMAP amplitudes and normal conduction velocities in all the nerves studied. Some markers of demyelination were also observed: temporal dispersion in the left ulnar nerve and only slightly prolonged minimal F wave latency in the contralateral ulnar nerve (32.1 ms, normal value ≤ 32 ms). Facial nerves were the most severely involved with reduced cMAP amplitudes (0.1 mV in the right one and 0.2 mV in the left one, normal value ≥ 1 mV) and prolonged distal latency in the left facial nerve (5.5 ms, normal value ≤ 4.2 ms). The spinal tap showed hyperproteinorrachia (1280 g/L), lymphocytic pleocytosis (10 cells/mmc), no oligoclonal bands and no signs of neoplastic infiltration. Laboratory tests were normal including anti-gangliosides antibodies, anti-acetylcholine receptor (AChR) and anti-muscle-specific kinase (MUSK) antibodies, anti-voltage-gated calcium channel antibodies and onconeural antibodies.

Based on the results of the spinal tap, we considered a Guillain-Barré syndrome (GBS). However, symptoms appeared and worsened in 6 weeks, so their evolution should be considered subacute. Moreover, only 2 months before the onset of neurological complications, she experienced hives on face and chest and headache that are classically reported as BRAF and MEK inhibitors irAE. Finally, she also had an excellent response to steroid treatment, normally ineffective in GBS.

On the whole, we diagnosed a sub-acute immune-mediated motor polyneuropathy with cranial nerves involvement related to targeted therapy.

Vemurafenib and Cobimetinib were stopped, and, considering that she had previously obtained benefit from steroid medication, we decided to treat her with IV methylprednisolone (1000 mg for 5 days) with a rapid recovery. In the following year, she could gradually taper oral prednisone, and she did not experience any relapse. No melanoma recurrence appeared.

### Patient 2

This 65-year-old man underwent hemicolectomy and partial ileal resection for a gastrointestinal stromal tumour (GIST). Afterwards he began oral adjuvant therapy with Imatinib (400 mg/day), a tyrosine kinase inhibitor (TKI) and the first drug used as targeted therapy. Few days after the first administrations, he developed neck muscle weakness, chewing and swallowing difficulties, so Imatinib treatment was temporary stopped for 2 days, with symptoms improvement. Subsequently, Imatinib was restarted, and 2 days later, the previous symptoms appeared again, associated with the onset of right eye ptosis. The neurological examination showed fluctuating and fatigable right eye ptosis, marked neck extensor weakness with head drop, dysphagia, dysphonia and masticatory weakness. Limb weakness was absent, and sensory examination was normal. Brain CT and Chest Radiograph did not show significant abnormalities. Blood test revealed CPK level slightly elevated (462 U/L). Because of the strong suspicion of myasthenic syndrome, we administered symptomatic therapy (pyridostigmine 60 mg, four times a day), and we planned five sections of plasma exchange. Despite these treatments, the patient rapidly worsened, and, after the second plasma exchange session, he developed acute respiratory failure. He was transferred to the Intensive Care Unit, and mechanical ventilation was supplied. In addition, steroid therapy was administered (methylprednisolone 120 mg/day). In the meanwhile, anti-AChR antibodies were tested and showed high-titre positivity, confirming myasthenia gravis. Anti-titin antibodies also resulted positive, while anti-MusK antibodies were negative. Total body CT excluded thymic disease and GIST metastases. After a course of intravenous immunoglobulins (IVIg) cycle (2 g/kg), the patient recovered. After hospital discharge, the patient performed 1 month of intensive rehabilitation, and his neurological examination returned completely normal. However, because of anti-AChR antibodies still show high titre positivity, steroid therapy was maintained over the following months even if gradually tapered.

## Discussion

In this work, we describe two new cases of oncological patients, who developed neuromuscular complications not related to ICPi administration but to other targeted therapies whose immune-mediated adverse events are much less known and described.

With regard to the first case, in literature, there are only few reported cases of bilateral facial palsy or acute polyradiculoneuropathy in patients treated with Vemurafenib- and Cobimetinib-combined treatment [5–9]. The distinctive trait of our patient is that she developed both the polyradiculoneuropathy and the bilateral cranial nerves involvement 3 years after the beginning of Vemurafenib and 1 year after the combined treatment, while the other cases developed polyradiculoneuropathy or bilateral facial palsy and generally few months after the start of therapy (4–9 weeks).

In general, for targeted therapy and for the most studied ICPi in particular, there is a wide variability from the first drug administration to irAE onset. IrAE may occur at any time and target multiple tissues, but generally cutaneous manifestations frequently appear in the first 2 weeks; then gastrointestinal side effects develop, usually within 6 weeks, followed by endocrine involvement (7–10 weeks) and respiratory system toxicity (8–14 weeks) [10]. However, very late reactions, even 1 year after stopping the treatment, may appear.

It is possible to hypothesize that the different organ involvement and the timing may depend both on tissue-specific immunity and on the different capacity of immune cells to reach different organs.

Moreover, in the last year, a possible link between early irAE onset and treatment efficacy emerged; however, late toxicities in responding, long-survivor patients have also been described [11].

Therefore, at the moment, why specific AE occur in specific patients, the different time interval and the link between irAE and treatment response are still a matter of debate.

It should be mentioned that the episodes of facial palsy and peripheral neuropathy reported in literature are mainly related to Vemurafenib treatment, while in our patient, they appeared only after Cobimetinib addition. It is like as Cobimetinib had strengthened not only anti-tumour effects but also the risks of AEs in a patient that until that moment was well tolerating the treatment.

Concerning the underlying mechanism for this specific AE, it has been demonstrated an increase in tumour-infiltrating lymphocytes in samples from patients after the beginning of BRAF inhibitor therapy, and the extent of infiltration correlates with tumour response. It is believed that this treatment leads to a suppression of the release of immunosuppressive factors from the melanoma cells and increases melanoma antigen expression leading to more effective T cell recognition [12]. We should remember that melanocytes and Schwann cells share the same origin from neural crest and surface molecules; for these reasons and for the marked steroid response, we think that the neuropathy was triggered by a mechanism of immune-mediated molecular mimicry [9].

With regard to the second case, to our knowledge, this is the only case of myasthenia gravis associated with Imatinib therapy. In literature there are only few reported cases concerning this topic. The first one refers to a patient with chronic myeloid leukaemia (CML) who developed seropositive MG 6 months after starting Nilotinib, a second-generation TKI. The patient was treated with pyridostigmine and prednisone with symptoms resolution and continued Nilotinib to achieve a major molecular response. The authors themselves were uncertain if the development of MG was related to Nilotinib, taking into account immunomodulatory effect reported by most of the TKI or to CML and related disturbed immune regulation [13].

The second report is about a man treated with Imatinib for CML who developed ocular MG with anti-AChR antibodies positivity after 4 months of therapy. A mediastinal mass was identified and resulted in thymic hyperplasia. The authors concluded that MG was unmasked by Imatinib, related to a disturbance of the immune function, but they could not rule out co-incidental occurrence of MG and LMC or a paraneoplastic manifestation of LMC [14]. Nevertheless, to our opinion, the presence of thymic hyperplasia should not be overlooked. In our case, the strict temporal association and the lack of thymic pathology strengthen Imatinib-MG association.

We also considered the possible association between GIST and MG as a paraneoplastic neurological syndrome (PNS). In this regard, there is a report of a newly diagnosed patient with seropositive MG who simultaneously underwent surgery for a gastric GIST, identified, but not removed, 2 years earlier. The authors themselves excluded a paraneoplastic origin, considering both the time course (generally, PNS precedes tumour diagnosis) and that PNS rarely associate with GIST [15]. In our case, MG appeared after GIST diagnosis and when the tumour had already been removed, allowing us to exclude a PNS. Moreover, paraneoplastic MG is mostly associated with thymoma.

Also in this case, an immune event can be hypothesized as the pathogenic mechanism at the basis of the development of MG following Imatinib treatment.

TKI show a broad spectrum of immunomodulatory, or “off-target”, effects, which allow long-term therapeutic efficacy that in many patients persists beyond treatment cessation. Among immune mechanism involved, it has been demonstrated that Imatinib, with both “on-target” and “off-target” pathways, inhibits tumour-infiltrating Treg lymphocytes, which have immunosuppressive functions, allowing cytotoxic T cell lymphocyte (CTL) relative expansion and anti-tumour suppression [16].

However, in normal conditions, Treg cells are essential for maintaining peripheral tolerance against self-antigens, and their dysfunction is implicated in the pathogenesis of different autoimmune diseases.

In patients with MG associated with thymoma, a decreased production of Treg cells has been described along with Treg cells subpopulations imbalance in respect to healthy controls. Therefore, a local disequilibrium of Treg and T effector cells is considered to be relevant for MG pathogenesis [17].

In these terms, we can hypothesize that Treg cells dysfunction is the common final pathway involved both in the pathogenesis of “classic” MG related to thymoma and MG related to Imatinib treatment.

In conclusion, we strengthen the relevance of neuromuscular complications in patients treated not only with the latest ICPi, but also with “older” and apparently better-known targeted therapies. Also in the latter cases, immune “off-target” mechanisms are involved, and consequences can be life-threatening if not promptly diagnosed and appropriately managed. Once the other diagnosis are excluded and an irAE is confirmed or strongly suspected, first line steroid treatment should be administered; if not sufficient, IVIg, plasma exchange or other “off-label” immunosuppressive treatment can be considered accordingly to patient’s need.
